# Effects of royal jelly supplementation on regulatory T cells in children with SLE

**DOI:** 10.3402/fnr.v60.32963

**Published:** 2016-11-24

**Authors:** Asmaa M. Zahran, Khalid I. Elsayh, Khaled Saad, Esraa M.A. Eloseily, Naglaa S. Osman, Mohamd A. Alblihed, Gamal Badr, Mohamed H. Mahmoud

**Affiliations:** 1 Clinical Pathology Department, South Egypt Cancer Institute, Assiut University, Assiut, Egypt; 2 Pediatric Department, Faculty of Medicine, Assiut University, Assiut, Egypt; 3 Medical Biochemistry Department, College of Medicine, Taif University, Taif, Kingdom of Saudi Arabia; 4 Zoology Department, Laboratory of Immunology & Molecular Physiology, Faculty of Science, Assiut University, Assiut, Egypt; 5 Deanship of Scientific Research, King Saud University, Riyadh, Kingdom of Saudi Arabia; 6 Food Science and Nutrition Department, National Research Center, Dokki, Cairo, Egypt

**Keywords:** royal jelly, regulatory T cells, children, systemic lupus erythematosus

## Abstract

**Background and objective:**

To our knowledge, no previous studies have focused on the immunomodulatory effects of fresh royal jelly (RJ) administration on systemic lupus erythematosus (SLE) in humans. Our aim was to study the effect of fresh RJ administration on the disease course in children with SLE with some immunological markers (CD4^+^ and CD8^+^ regulatory T cells and T lymphocytes apoptosis).

**Methods:**

This was an open-label study in which 20 SLE children received 2 g of freshly prepared RJ daily, for 12 weeks.

**Results:**

The percentages of CD4^+^ CD25^+high^ FOXP3^+^cells (CD4^+^ regulatory T cells) and CD8^+^CD25^+high^ FOXP3^+^cells (CD8^+^ regulatory T cells) were significantly increased after RJ treatment when compared with baseline values. Apoptotic CD4 T lymphocytes were significantly decreased after RJ therapy when compared with baseline values and the control group.

**Conclusion:**

This is the first human study on the effect of RJ supplementation in children with SLE. Our results showed improvements with 3-month RJ treatment with regard to the clinical severity score and laboratory markers for the disease. At this stage, it is a single study with a small number of patients, and a great deal of additional wide-scale randomized controlled studies are needed to critically validate the efficacy of RJ in SLE.

Systemic lupus erythematosus (SLE) is a common autoimmune disease characterized by abnormal T- and B-cell activation, cytokines disorders, and considerable production of autoantibodies (1). Despite central mechanisms of tolerance, some T cells recognizing self-antigens are released into the periphery. One of the mechanisms employed to eliminate or control these potentially damaging cells is regulatory T cells (Tregs). A number of immune Tregs have been described (2). CD4^+^ Tregs play an important role in the maintenance of peripheral tolerance and are characterized by the expression of the CD25 surface marker and the transcription factor forkhead box protein 3 (FOXP3), making up to 5–10% of the normal CD4^+^ T-cell population. Abrogation of CD4 Tregs development and/or peripheral function may cause autoimmune diseases (3).

CD8^+^CD25^+^FOXP3^+^ T cells with regulatory function in maintaining self-tolerance have been identified and can be generated by continuous antigen stimulation. Human CD8^+^ Tregs are implicated in autoimmune disorders: multiple sclerosis and inflammatory bowel disease (2, 4). Suppressive CD8 Tregs appear after T-cell receptor stimulation, suppressing cellular proliferation of CD4^+^ naïve and effector T cells via cell–cell contact lysis or soluble factors like interleukin (IL) 10 and transforming growth factor beta (TGF-β). CD8 Tregs have suppressive ability typically associated with CD4^+^ Tregs. Interaction between subsets of Tregs that protect against autoimmune diseases remains unclear (4, 5). FOXP3-expressing CD8^+^ T cells proved vital for CD4^+^CD25^+^ Tregs induced by a tolerogenic peptide to suppress murine lupus (5).

Apoptosis is a programmed cell death and, normally, the resulted apoptotic cells are removed by phagocytes. Failure to remove apoptotic bodies leads to the release of autoantigens which may induce autoimmunity. Increased lymphocyte apoptosis is found to be part of the pathogenesis of SLE. Studies in human SLE have revealed increased lymphocyte apoptosis and defects in macrophage removal of apoptotic cells (6–8).

Royal jelly (RJ) is a creamy product secreted by young nurse worker bees (*Apis mellifera*), and it is synthesized in the hypopharyngeal and mandibular glands. RJ comprises 60–70% water, 10–12% carbohydrates, 12–15% proteins and 3–7% lipids. Based on unique composition of RJ, recently *in vivo* and *in vitro* studies focused on its health-promoting characteristics such as hypotensive, anti-hypercholesterolemia, anti-inflammatory, antitumor, antioxidant and weight management effects and immunomodulatory properties (9, 10). The immunomodulatory properties were approved in animal studies of SLE, as RJ administration to mice caused a significant decrease in the serum level of IL-10 and in the autoantibodies against single-stranded deoxyribonucleic acid (ssDNA), double-stranded deoxyribonucleic acid (dsDNA), and erythrocytes, as well as a reduction in the number of splenic autoreactive B cells (9). We hypothesized that RJ has a role in controlling the disease activity of SLE through its immunomodulatory effect on Tregs. To our knowledge, no previous studies have focused on the immunomodulatory effect of RJ administration on SLE patients in humans. Our aim was to study the effect of RJ supplementation on the disease course of pediatric SLE with some immunological markers (the CD4 and CD8 Tregs) and lymphocytes apoptosis.

## Patients and methods

The Ethical Committee of Assiut University, Assiut, Egypt, approved the study. Participants were given a complete description of the study, and a written informed consent in accordance with Assiut University Ethical Committee guidelines was taken from parents of all the participants. The participants can leave the study at any time. The work has been carried out in accordance with the code of ethics of the World Medical Association (Declaration of Helsinki) for experiments involving humans.

### Study setting

The study was conducted in the Pediatric Immunology and Rheumatology Unit, Assiut University Children Hospital, Assiut, Upper Egypt, from June 2015 to January 2016.

### Study subjects

A total of 20 SLE children, with 18 girls and 2 boys, who regularly followed up at Assiut University Children Hospital were included in this study. The inclusion criteria included children <18 years old, who are fulfilling at least four of the American College of Rheumatology Revised Criteria for SLE (11), and 20 sex- and age-matched healthy controls were enrolled in this study.

Exclusion criteria were children who were known to have hypersensitivity to honey, and children with congenital abnormalities of respiratory and GI systems. In addition, children younger than 1 year who had severe malnutrition and who had taken any recent vaccination were excluded.

### Study design

This was an open-label study in which SLE children received 2 g of freshly prepared RJ daily, for 12 weeks. RJ was advised to be taken on an empty stomach at least 30 min before meals. All children in the study were assessed by taking medical history, performing clinical examinations. Serologic records were collected at the time of enrollment, and after 3 months of the RJ therapy. Anti-dsDNA antibodies, antinuclear antibodies (ANA), C3, C4, complete blood count, serum albumin, serum creatinine, serum urea, erythrocyte sedimentation rate (ESR), C-reactive protein (CRP), protein in urine, and urine red blood cells (RBCs) were done for all SLE patients before and after RJ therapy. Immunological markers (the CD4 and CD8 Tregs) and lymphocytes apoptosis were done for healthy controls and SLE children at baseline and at the end of the study.

### Flow cytometric detection of apoptosis of lymphocytes

**Fig. 1:**
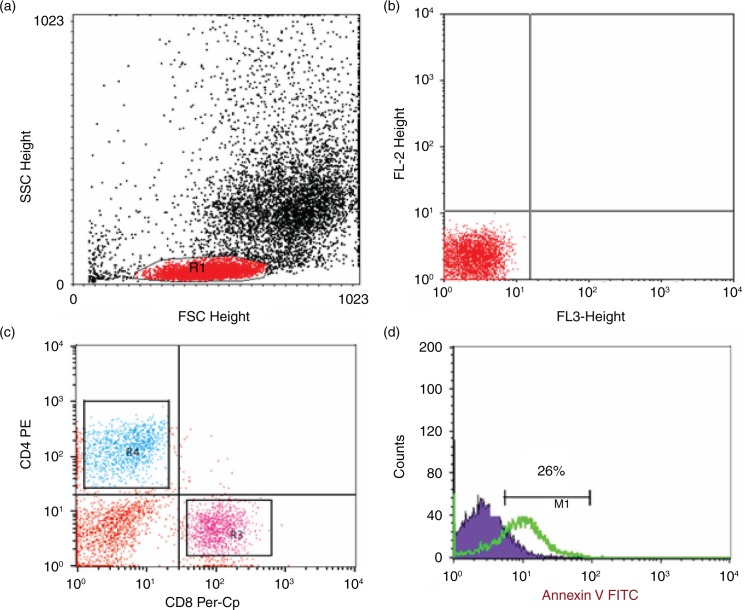
Flow cytometric analysis of T lymphocytes apoptosis. (a) Forward and side scatter histogram was used to define the lymphocytes. (b, c) The expression of CD8 and CD4 was assessed in lymphocytes population compared with the negative isotype control. The T-helper cells (CD4^+^) and T cytotoxic cells (CD8^+^) were gated for further analysis of annexin V expression. (d) Example of annexin V expression on T cells (CD4^+^ or CD8). The positivity was defined as fluorescence (green histogram) higher than that of the isotype control (blue histogram).

Apoptosis was measured by the annexin V–fluoroisothiocyanate (FITC) binding assay according to the manufacturer's instructions (BD Biosciences, San Jose, CA, USA). Fifty microliters of whole blood was stained with 5 µl of peridinium-chlorophyll-protein (Per-CP)-conjugated anti-CD8 and phycoerythrin (PE)-conjugated anti-CD4, washed twice with 2 ml of phosphate-buffered saline (PBS), and RBCs were lysed. The cells were washed and resuspended in 100 µl of the annexin V-conjugate binding buffer to which 5 µl of FITC-conjugated annexin V was added. The mixture was incubated in dark at room temperature for 15 min, after which 400 µl of the binding buffer was added and 10,000 cells were acquired and analyzed by FACSCalibur flow cytometry. Anti-human immunoglobulin G (IgG) was used as an isotype-matched negative control for each sample. Forward and side scatter histogram was used for lymphocytes populations. Then, the percentages of CD4^+^ (T-helper lymphocytes) and CD8^+^ (T cytotoxic lymphocytes) were assessed in the lymphocyte populations. Then, the expression of annexin V in CD4^+^ and CD8^+^ lymphocytes was detected (Fig. 1).

### Flow cytometric detection of regulatory T lymphocytes

**Fig. 2:**
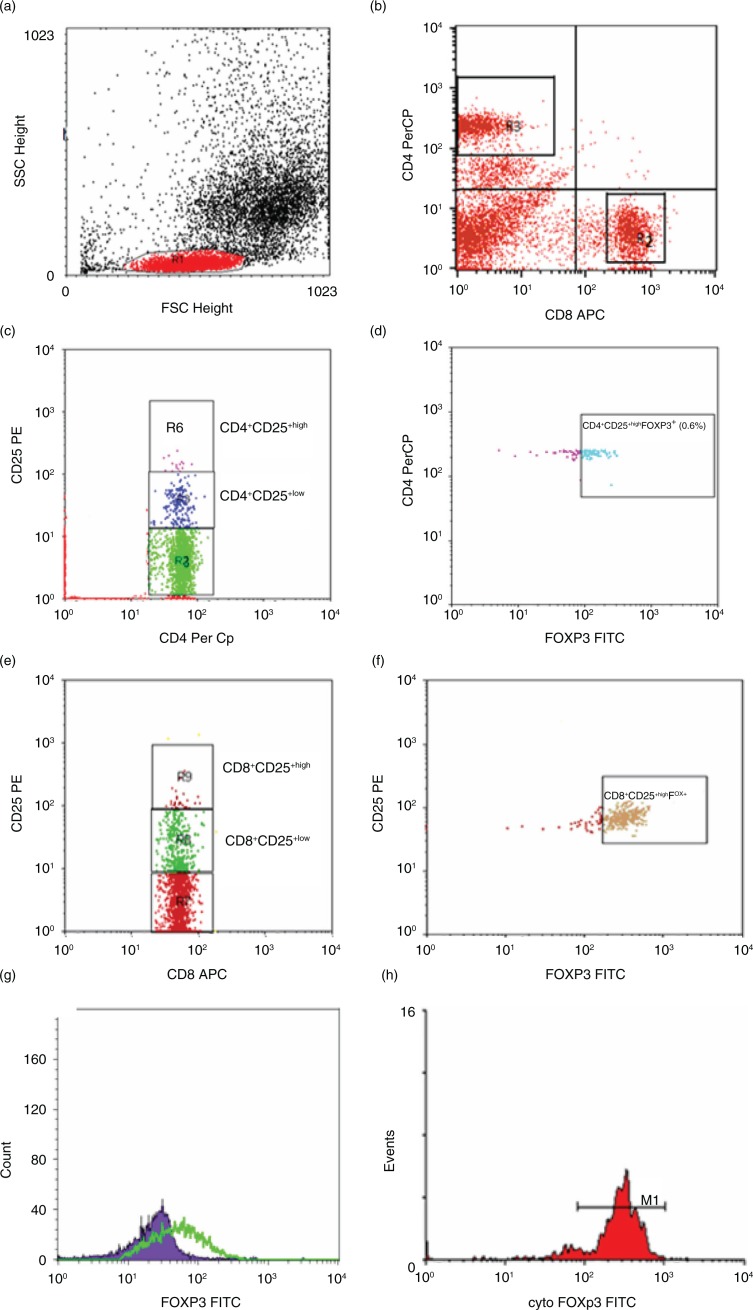
Flow cytometric detection of regulatory T cells. (a) Forward and side scatter histogram was used to define the lymphocytes population (R1). (b) The expression of CD8^+^ (R2) and CD4^+^ (R3) was assessed in lymphocytes population. (c) The expression of CD25 in CD4^+^ was detected and compared with the negative isotype control (not shown), and different gates were drowned to define CD4^+^CD25^+low^ cells (R5) and CD4^+^CD25^+High^ cells (R6). (d) The percentage of CD4^+^CD25^+high^ FOXP3^+^cells (CD4^+^ regulatory T cells) was determined. (e, f) The detection of CD8^+^ regulatory T cells in CD8^+^ cells. The expression of CD25 in CD8^+^ was detected. Then, CD8^+^CD25^+low^ cells (R8), and CD8^+^CD25^+High^ (R9) cells and CD8^+^CD25^+high^ FOXP3^+^cells (CD8^+^ regulatory T cells) were determined. (g, h) The expression of FOXP3^+^ in CD4^+^CD25^+High^ cells CD8^+^CD25^+high^ cells.

Tregs in whole blood samples were enumerated using FITC-conjugated anti-FOXP3 (eBioscience, San Diego, CA, USA), PE-conjugated anti CD25 (IQ Product, Groningen, the Netherlands), Per-CP-conjugated anti-CD4, and allophycocyanin (APC)-conjugated anti-CD8 (Becton Dickinson, Bioscience, USA). Fifty microliters of blood sample was incubated with 5 µl of CD4, CD8, and CD25 for 15 min at room temperature in the dark. Following incubation, RBC lysis, washing with PBS, addition of fixed solution to fix the cells, and incubation for 10 min were done. After incubation, cells were washed with PBS, and then permeabilized solution and 5 µl of FOXP3 were added and incubated for 30 min at room temperature. After one wash, the cells were resuspended in PBS. Flow cytometric analysis was done by FACSCalibur flow cytometry with CellQuest software (BD Biosciences). Anti-human IgG was used as an isotype-matched negative control for each sample. Forward and side scatter histogram was used to define the lymphocytes population (R1). Then, the percentages of CD4^+^ (T-helper cells) and CD8^+^ (T cytotoxic cells) were assessed in lymphocytes population. Total CD4^+^CD25^+^, CD4^+^CD25^+low^ and CD4^+^CD25^+High^ (defined as the population of CD4-positive T cells whose CD25 expression exceeded the level of CD25 positivity seen in the CD4-negative T cells), and CD4^+^CD25^+High^ FOXP3^+^Tregs were evaluated as a percentage of CD4^+^. Also, total CD8^+^CD25^+^, CD8^+^CD25^+intermediate (low)^ and CD8^+^CD25^+High^, and CD8^+^CD25^+High^ FOXP3^+^Tregs were evaluated as a percentage of CD8^+^. The expression of FOXP3^+^ in CD4^+^CD25^+high^ and in CD8^+^CD25^+high^ cells was expressed as a geometric mean of fluorescence intensity (MFI) as shown in Fig. 2.

### Outcome assessment

SLE children in our study were followed up for 3 months. Disease activity was assessed by SLE Disease Activity Index (SLEDAI). Active disease was defined as SLEDAI >4 (12). Regarding the clinical assessment, each patient served as his or her own control, and differences after 12 weeks of RJ therapy were calculated and compared with each patient's baseline values (at Week 0). Caregivers of participating children received a weekly telephone call from the research team reminding them to administer the therapy. They were not allowed to change the provided dose or to add any supplements throughout the study period. At follow-up visits, the parents were asked to report any difficulties or adverse effects during the study period. Side effects were recorded throughout the study and were assessed using a checklist every month. With regard to serologic, laboratory, and flow cytometric records, the patients were compared before and after RJ with healthy controls.

## Statistical analysis

Data analysis was performed with the Statistical Package for Social Sciences (SPSS version 17). Data are expressed as mean ± standard deviation (SD) for all parameters. Because of the small sample size and a propensity for outliers in some of the variables, statistical differences between the groups were examined using the Mann-Whitney test and Wilcoxon test. Spearman's correlation was used to determine the correlation between studied parameters. A value of *p*≤0.05 denoted a statistically significant difference.

## Results

Table 1 shows some demographic characteristics of SLE children. The mean age of the patients was 9.4±3.7 years (range 7–16 years) with a mean disease duration of 3.5±2.8 years (range 1–8 years). The mean SLEDAI score was 11.3±7.6 (range 2–31). Fifty percent of the patients were positive for anti-dsDNA and ANA antibodies. All clinical characteristics of SLE patients were summarized in Table 1.

**Table 1:** Baseline clinical data of all studied SLE children and controls

Parameter	SLE patients (20)	Control (20)	*p*
Age (years)			
Range	7–16	7–16	NS
Mean±SD	9.4±3.7	9.0±4.5	NS
Disease duration (years)	4.5±2.8	–	–
Male/female	2/18	2/18	NS
SLEDAI			
Range	2–31	–	–
Mean±SD	11.3±7.6	–	–
ACR criteria of SLE: no. (%)			
Malar rash	7 (35%)	–	–
Discoid rash	2 (10%)	–	–
Photosensitivity	2 (10%)	–	–
Oral ulcers	1 (5%)	–	–
Arthritis	11 (55%)	–	–
Serositis	5 (25%)	–	–
Renal disorders	8 (40%)	–	–
Neuropsychiatric disorders	9 (45%)	–	–
Hematological disease	13 (65%)	–	–
Positive antinuclear antibodies	10 (50%)	–	–
Positive anti-dsDNA antibodies	10 (50%)	–	–
Other clinical data			
Fatigue	16 (80%)	–	–
Pulmonary disorders	3 (15%)	–	–

ACR, American College of Rheumatology; NS, not significant; SD, standard deviation; SLE, systemic lupus erythematosus; SLEDAI, Systemic Lupus Erythematosus Disease Activity Index.

Table 2 shows laboratory data of SLE children and SLEDAI before and after RJ supplementation and the controls. SLEDAI score was significantly improved after 3 months of RJ therapy (mean±SD, 11.3±7.6 vs. 8.3±6.1; *p*=0.01). Complement C3 (*p*=0.009) and C4 (*p*=0.014) levels were significantly increased after RJ therapy.

**Table 2:** Some laboratory data and SLEDAI in SLE patients before and after Royal Jelly supplementation and the controls

	SLE patients before RJ supplementation (20)	SLE patients after RJ supplementation (20)	Controls (20)	*p*^a^	*p*^b^	*p*^c^
SLEDAI	8.3±4.1	3.3±2.9	ND	–	0.01	–
C3 (mg/dl)	0.44±0.09	1.1±0.13	ND	–	0.009	–
C4 (mg/dl)	0.17±0.030	0.82±0.17	ND	–	0.014	–
Hemoglobin (g/dl)	9.71±0.51	11.57±0.26	12.3±0.28	0.001	0.025	NS
Platelets (10^3^/µl)	252.06±47.69	270.89±28.7	290±24.5	NS	NS	NS
WBC (10^3^/µl)	5.32±0.54	7.01±0.83	7.29±0.41	0.004	0.018	NS
Albumin (g/dl)	2.93±0.21	4.02±0.13	4.03±0.09	<0.001	0.001	NS
Creatinine (mg/dl)	2.99±0.38	1.32±0.22	0.73±0.06	<0.001	0.002	0.004
Urea (mg/dl)	151.86±29.6	55.15±4.75	19.51±2.37	<0.001	0.003	0.001
ESR (mm/h)	77.68±10.12	20.22±2.4	9.2±0.75	<0.001	0.004	0.001
Protein in urine (mg/dl)	369.6±123.17	23.11±4.8	Negative	–	0.041	–
Anti-dsDNA	0.78±0.14	0.71±0.09	Negative	–	NS	–
CRP	3.68±0.2	1.88±0.18	Negative	–	<0.001	–
Urine RBCs	3.43±0.35	2.87±0.12	Negative	–	NS	–

Data are represented as means±SD. *P*≤0.05 is significant. CRP, C-reactive protein; ESR, erythrocyte sedimentation rate; ND, not determined; NS, not significant; RBC, red blood cells; RJ, royal jelly; SLE, systemic lupus erythematosus; SLEDAI, Systemic Lupus Erythematosus Disease Activity Index; WBC, white blood cells;

a SLE patients before RJ supplementation versus controls (Mann–Whitney *U*-test)

b SLE patients before RJ supplementation versus SLE patients after RJ supplementation (Wilcoxon test)

c SLE patients after RJ supplementation versus controls (Mann–Whitney *U*-test).

Regarding the hematological parameters, there were no statistically significant differences in platelet counts in the peripheral blood among patients and the controls. The mean hemoglobin level was significantly improved after 3 months of RJ therapy (mean±SD, 9.71±0.51 vs. 11.57±0.26; *p*=0.025).

White blood cell counts, serum albumin, serum creatinine, serum urea, ESR, CRP, and protein in urine were significantly improved after 3 months of RJ therapy; however, no significant changes were observed in urine RBCs and anti-dsDNA antibodies at the end of the therapy (Table 2).

The percentages of CD4^+^CD25^+high^ and CD4^+^CD25^+high^ FOXP3^+^ cells (CD4^+^ regulatory T cells) were significantly increased after RJ treatment versus baseline value, however, the values prior and after RJ therapy were statistically significantly lower than control group (Table 3).

**Table 3:** Regulatory T cells in patients with systemic lupus erythematosus before and after royal jelly supplementation and the controls

	SLE patients before RJ supplementation (20)	SLE patients after RJ supplementation (20)	Controls (20)	*p*^a^	*p*^b^	*p*^c^
CD4^+^ regulatory T cells	1.32±0.25	1.74±0.28	2.24±0.52	<0.001	0.001	0.001
CD8^+^ regulatory T cells	0.93±0.37	1.58±0.84	2.01±0.98	<0.001	0.003	NS
MFI of FOXP3^+^ expression in CD4^+^CD25^+High^	102.98±6.94	118.16±29.96	149.72±19.3	<0.001	0.032	0.001
MFI of FOXP3^+^ expression in CD8^+^CD25^+High^	96.85±12.51	113.09±14.49	116.98±33.54	0.016	0.01	NS

Data are represented as means±SD. *P*≤0.05 is significant. FOXP3, forkhead box protein 3; MFI, mean of fluorescence intensity; NS, not significant; RJ, royal jelly; SLE, systemic lupus erythematosus;

a SLE patients before RJ supplementation versus controls (Mann–Whitney *U*-test)

b SLE patients before RJ supplementation versus SLE patients after RJ supplementation (Wilcoxon test)

c SLE patients after RJ supplementation versus controls (Mann–Whitney *U*-test).

The frequencies of CD8^+^CD25^+high^ and CD8^+^CD25^+high^ FOXP3^+^cells (CD8^+^ regulatory T cells) were significantly increased after RJ treatment versus baseline value. The percentages of CD8^+^CD25^+high^ and CD8^+^ regulatory T cells at baseline were significantly lower than control group. After RJ supplementation, there were no significant difference between patients and controls (Table 3).

Table 4 shows that patients with SLE (both before and after RJ treatment) had significantly lower counts of CD4 lymphocytes versus the normal control group. There was also significantly lower CD4^+^/CD8^+^ cell ratio in patients than the controls. The percentage of CD4^+^ T lymphocytes was significantly increased after RJ treatment versus baseline value (mean%±SD%, 31.06%±1.23% vs. 37.48%±1.54%; *p*=0.008). This was not true for CD8^+^ T lymphocytes as it did not show any significant changes with RJ treatment or any difference between the SLE children and normal control group.

**Table 4:** T lymphocytes and their apoptosis in children with SLE before and after royal jelly supplementation and the controls

	SLE patients before RJ supplementation (20)	SLE patients after RJ supplementation (20)	Controls (20)	*p*^a^	*p*^b^	*p*^c^
CD4 T lymphocytes	31.06±1.23	37.48±1.54	46.47±2.85	0.002	0.008	0.023
CD8 T lymphocytes	29.11±1.42	28.54±0.92	31.12±1.12	NS	NS	NS
CD4/CD8 ratio	1.25±0.06	1.53±0.09	1.69±0.107	0.001	0.02	NS
Apoptotic CD4 T lymphocytes	32.35±3.75	8.45±0.23	8.09±0.26	0.001	0.018	NS
Apoptotic CD8 T lymphocytes	9.53±0.47	9.31±0.41	8.29±0.40	NS	NS	NS

Data are represented as means±SD. *P*≤0.05 is significant. NS, not significant; RJ, royal jelly; SLE, systemic lupus erythematosus;

a SLE patients before RJ supplementation versus controls (Mann–Whitney *U*-test)

b SLE patients before RJ supplementation versus SLE patients after RJ supplementation (Wilcoxon test)

c SLE patients after RJ supplementation versus controls (Mann–Whitney *U*-test).

With regard to the apoptosis data, at baseline, SLE patients exhibited a significant increase in the percentage of apoptotic CD4^+^ T lymphocytes (mean%±SD%, 32.35%±3.75% vs. 8.09%±0.26%; *p*=0.001) and an insignificant increase in the percentage of apoptotic CD8 T lymphocytes (mean%±SD%, 9.53%±0.47% vs. 8.29%±0.40%; *p*=0.07) when compared with healthy children (Table 4). Apoptotic CD4^+^ T lymphocytes were significantly decreased after RJ therapy (mean%±SD%, 32.35%±3.75% vs. 8.45±0.23%; *p*=0.018) when compared with baseline values (Table 4). The frequency of apoptotic CD8^+^ T lymphocytes was decreased after RJ therapy; however, this was statistically insignificant (mean%±SD%, 9.53%±0.47% vs. 9.31%±0.41%; *p*=0.678). The frequencies of both apoptotic CD4 and CD8 T cells were comparable between SLE patients after RJ therapy and controls (Table 4).

## Adverse events

RJ supplementation was generally well tolerated. We reported temporally associated side effects in four patients (20%) during the study period: skin rash (one patient), itching (one patient), and stomach pain (two patients). All side effects were mild and transient, and all patients continued the 3-month RJ treatment.

## Discussion

SLE is a common autoimmune disease characterized by abnormal immune regulation and the production of autoantibodies to self-nuclear, cytoplasmic, and cell surface molecules with the resulting generalized multisystem chronic autoimmune inflammatory disease (1, 2, 13). RJ is known to have multipotent health beneficial actions; the immunomodulatory effects in particular are being increasingly proven in the literature (9). RJ was found to stimulate antibody production and immunocompetent cell proliferation in mice (14). Furthermore, RJ was found to have anti-inflammatory effects through different mechanisms, one was due to the inhibition of proinflammatory cytokine production by macrophages (15). In addition, RJ was suggested to modulate the immune responses in rats through affecting their dendritic cells by its fatty acid contents (16). RJ was found to suppress type-I allergic reactions and was associated with the restoration of macrophage function and Th1/Th2 cytokine responses (17). RJ was also found to inhibit the development of atopic dermatitis-like lesions in mice (18).

Based on these immunomodulatory properties, RJ was challenged in some *in vivo* and animal studies of autoimmune diseases, and the results were promising. With regard to SLE, there is only one animal study that explored this effect. In an animal model of SLE in New Zealand Black×New Zealand White F1 mice that genetically exhibit many manifestations similar to human SLE, RJ oral administration showed a significant delay in the onset of the disease and its active progression. Mice that received RJ showed decreased proteinuria and a prolongation of lifespan. RJ caused a significant decrease in the serum level of IL-10, and in the autoantibodies against ssDNA, dsDNA, and erythrocytes, as well as a reduction in the number of splenic autoreactive B cells (9).

In our cohort of children with SLE, before treatment there was an observed imbalance between CD4^+^ and CD8^+^ lymphocytes; this may be explained by the immune dysregulation in cases of SLE. Our results showed that patients with SLE (both before and after RJ treatment) had significantly lower percentages of CD4^+^ lymphocytes versus the normal control group. There was also significantly lower CD4^+^/CD8^+^ cell ratio in patients than the controls. The frequency of CD4^+^ T lymphocytes was significantly increased after RJ treatment versus baseline value. This was not true for CD8^+^ T lymphocytes as it did not show any significant changes with RJ treatment or any difference between the SLE children and normal control group.

Our results are in accordance with what was found by McInerney et al. (19) who examined the levels and function of peripheral blood immunoregulatory T-cell subpopulations in SLE. They found normal percentages of CD8^+^ T cells in peripheral blood in all SLE patients. Interestingly, about CD4^+^, they found about half of the SLE patients had markedly depressed CD4^+^ cell levels and in turn significantly lower CD4^+^/CD8^+^ cell ratio, whereas the remaining half of the patients had normal levels of CD4^+^ cells (normal CD4^+^/CD8^+^ cell ratio) (19).

FOXP3 is a key transcription factor that is critically important in controlling Treg development and function. There are many evidence that FOXP3 is involved in the pathogenesis of autoimmune diseases. In our previous study, we found that CD4^+^CD25^+High^ and CD4^+^ CD25^+High^ FOXP3^+^ were significantly decreased in total lymphocytes and in CD4^+^ cells in the recently diagnosed diabetes type I pediatric patients (20). Moreover, in a mouse model (collagen induced) of rheumatoid arthritis, FOXP3 transduction led to inhibition of T-cell activation and attenuation of the cell proliferation. It decreased IL-2 and interferon (IFN)-gamma expression, and increased IL-10 expression in activated CD4^+^CD25^−^ T cells. FOXP3-transduced CD4^+^CD25^−^ T cells attenuated proliferation of activated CD4^+^CD25^−^ T cells. FOXP3 transduction delayed disease incidence remarkably and alleviated autoimmune symptoms of collagen-induced arthritis mice (21).

In this study, we found that the children with SLE (in both before and after RJ treatment) had significantly lower levels of CD4 regulatory T cells and FOXP3 expression in CD4^+^CD25^+High^ versus normal control group. Also, the levels of CD4^+^ regulatory T cells and FOXP3 expression were negatively correlated with disease activity as detected by SLEDAI. This is in accordance with Hu et al. (1) and Lyssuk et al. (22) who showed that the CD4^+^ regulatory T cells and FOXP3 levels in SLE patients were significantly lower than normal controls. Also, they found a significant negative correlation between Tregs level and the disease activity index (SLEDAI scores). CD4^+^ regulatory T cells is suggested to play an important role in the pathogenesis of SLE (1, 22). On the contrary, there has been very contradicting results between studies of CD4^+^FOXP3^+^ cells in SLE. Barath et al. (23) found decreased CD4^+^CD25^+^FOXP3^+^ cells in SLE without any correlation with disease activity. Other studies have reported normal values for CD4^+^CD25^+^FOXP3^+^ cells (24, 25). Surprisingly, other researchers have reported increased percentages of CD4^+^ FOXP3^+^ in lupus and found that this result correlated with disease activity (26).

The significant increase in the level of CD4^+^ regulatory T cells in our SLE patients after treatment with RJ may be explained by its immunomodulatory effects through its ability to increase proliferation of immune competent cells (14). It is also found to modulate the immune responses in rats through affecting their dendritic cells through affecting their fatty acid content (16). A recent study has shown a special active substance that exists only in RJ that is called ‘hydroxyl-2-decenoic acid’. It was found to promote the growth of T lymphocyte subsets and IL-2 production (27). In an animal model of SLE, oral administration of RJ showed significant delay in the disease onset and its active progression. In our patients, there was a decrease in disease activity after administration of RJ as detected by SLEDAI. Our patients also showed a negative correlation between the disease activity and CD4 and FOXP3, which may suggest that the rate of increase of the regulatory T cells has an impact on decreasing the disease activity. La Cava et al. (28) demonstrated that the production of autoantibodies by B cells in SLE patients could be interrupted via induction of regulatory T cells, and the regulatory T cells could inhibit the production of dsDNA antibodies by B cells via cell contact inhibition induced by membrane-bound TGF-β and Glucocorticoid-induced tumour necrosis factor receptor (GITR) molecules.

The CD8^+^ regulatory T cells interact with effector cells significantly, limiting immune response (1). The decreased level of CD8^+^ regulatory T cells in our patients before treatment indicates a poor ability of these cells to suppress the autoreactive T cells in SLE patients. Also, CD8^+^ regulatory T cells are essential for CD4^+^CD25^+^ regulatory T cell functions in the suppression of autoimmunity (29). This contributed to the ongoing immune dysregulation in our patients. After treatment with RJ, the level of CD8^+^ regulatory T cells was significantly increased and became non-significantly different from that of the controls. This can be explained by the effect of hydroxy-2-decenoic acid in promoting IL-2. CD8^+^ regulatory T cells expand in response to IL-2 treatment and can be characterized phenotypically and functionally, at steady state and under IL-2 stimulation. CD8^+^ regulatory T cells are highly suppressive and responsive to IL-2 (30).

Apoptosis is a programmed cell death, and normally the resulted apoptotic cells are removed by phagocytes. Failure to remove apoptotic bodies leads to the release of autoantigens, which may induce autoimmunity (7, 8). Our study revealed a significantly higher frequency in CD4^+^ T cells apoptosis, which could explain the reduction of CD4^+^ T cells in our patients with SLE. Increased lymphocyte apoptosis is found to be part of the pathogenesis of SLE. Studies in human SLE have revealed increased lymphocyte apoptosis and defects in macrophage removal of apoptotic cells, although the mechanism by which the macrophages are defective in removal of apoptotic cells is not yet clear. Taylor et al. (31) found that complement deficiency may be involved in this process. Ren et al. (8) have shown that macrophages from SLE patients were less capable of ingesting apoptotic neutrophils and that this was secondary to the presence or lack of serum factors rather than an intrinsic defect of the macrophages. Jin et al. (32) found that the macrophages activity was actually increased in SLE in response to increased lymphocytes apoptosis. This was confirmed by the finding that serum levels of neopterin and IFN-γ was increased in SLE. Also, they found that neopterin correlated positively with the increased number of apoptotic lymphocytes and the overall SLE disease activity. Moreover, they found that lymphocyte apoptosis and increased macrophages activity were correlated with the disease activity.

With regard to the effect of RJ treatment on lymphocytes apoptosis in our study, we found that the apoptotic CD4^+^ T lymphocytes showed significant reduction to about its quarter of the baseline values before treatment to values about normal as the healthy control group. Again in correlation with the previous CD8^+^ T lymphocytes count, the changes in apoptotic CD8^+^ T lymphocytes were insignificant between patients and controls.

## Conclusion

To the best of our knowledge, this is the first human study on the effect of RJ supplementation in children with SLE. Our results showed improvements with 3-month RJ treatment in regard to the clinical severity score and laboratory markers for the disease. At this stage, it is a single study with a small number of patients, and a great deal of additional wide-scale randomized controlled studies are needed to critically validate the efficacy of RJ in SLE.

## Limitations of the study and the impact on future research

There were some limitations of this study. First, this was an open, uncontrolled study. Second, we investigated a small group of children with SLE. Further randomized controlled longitudinal studies with a larger sample size are warranted to confirm our findings and to explore the mechanism of action of RJ in SLE. SLE patients have higher serum levels of IL-17 than healthy controls. Moreover, the frequency of IL-17-producing T cells is increased in peripheral blood of SLE patients (33). We recommend to analyze the regulation of IL-17 in context to RJ therapy in patients with SLE in the future studies.
